# Effect of rosuvastatin versus atorvastatin on new-onset diabetes mellitus in patients treated with high-intensity statin therapy for coronary artery disease: a post-hoc analysis from the LODESTAR randomized clinical trial

**DOI:** 10.1186/s12933-024-02386-w

**Published:** 2024-08-07

**Authors:** Sung-Jin Hong, Yong-Joon Lee, Woong Chol Kang, Bum-Kee Hong, Jong-Young Lee, Jin-Bae Lee, Tae-Hyun Yang, Junghan Yoon, Seung-Jun Lee, Chul-Min Ahn, Jung-Sun Kim, Byeong-Keuk Kim, Young-Guk Ko, Donghoon Choi, Yangsoo Jang, Myeong-Ki Hong, for the LODESTAR investigators

**Affiliations:** 1 Severance Hospital, Yonsei University College of Medicine, Seoul, Korea; 2 Gachon University College of Medicine, Incheon, Korea; 3 Gangnam Severance Hospital, Seoul, Korea; 4 Kangbuk Samsung Hospital, Sungkyunkwan University School of Medicine, Seoul, Korea; 5 Daegu Catholic University Medical Center, Daegu, Korea; 6 Inje University Busan Paik Hospital, Busan, Korea; 7 Wonju Severance Christian Hospital, Wonju, Korea; 8 CHA University College of Medicine, Seongnam, Korea; 9 Division of Cardiology, Severance Hospital, Yonsei University College of Medicine, 50-1 Yonsei- ro, Seodaemun-gu, Seoul, 03722 South Korea

**Keywords:** Statin, Coronary artery disease, Diabetes mellitus

## Abstract

**Background:**

The impact of rosuvastatin versus atorvastatin on new-onset diabetes mellitus (NODM) among patients treated with high-intensity statin therapy for coronary artery disease (CAD) remains to be clarified. This study aimed to evaluate the risk of NODM in patients with CAD treated with rosuvastatin compared to atorvastatin in the randomized LODESTAR trial.

**Methods:**

In the LODESTAR trial, patients with CAD were randomly assigned to receive either rosuvastatin or atorvastatin using a 2-by-2 factorial randomization. In this post-hoc analysis, the 3-year incidence of NODM was compared between rosuvastatin and atorvastatin treatment in the as-treated population with high-intensity statin therapy as the principal population of interest.

**Results:**

Among 2932 patients without diabetes mellitus at baseline, 2377 were included in the as-treated population analysis. In the as-treated population with high-intensity statin therapy, the incidence of NODM was not significantly different between the rosuvastatin and atorvastatin groups (11.4% [106/948] versus 8.8% [73/856], hazard ratio [HR] = 1.32, 95% confidence interval [CI] = 0.98 to 1.77, *P* = 0.071). When the risk of NODM with rosuvastatin versus atorvastatin was assessed according to the achieved low-density lipoprotein cholesterol (LDL-C) level, the risk of NODM began to increase at a LDL-C level below 70 mg/dL. The incidence of NODM was significantly greater in the rosuvastatin group than it was in the atorvastatin group when the achieved LDL-C level was < 70 mg/dL (13.9% versus 8.0%; HR = 1.79, 95% CI 1.18 to 2.73, *P* = 0.007).

**Conclusions:**

Among CAD patients receiving high-intensity statin therapy, the incidence of NODM was not significantly different between rosuvastatin and atorvastatin. However, a drug effect of the statin type on NODM was observed when the achieved LDL-C level was < 70 mg/dL.

**Trial registration:**

ClinicalTrials.gov, Identifier: NCT02579499.

**Graphical abstract:**

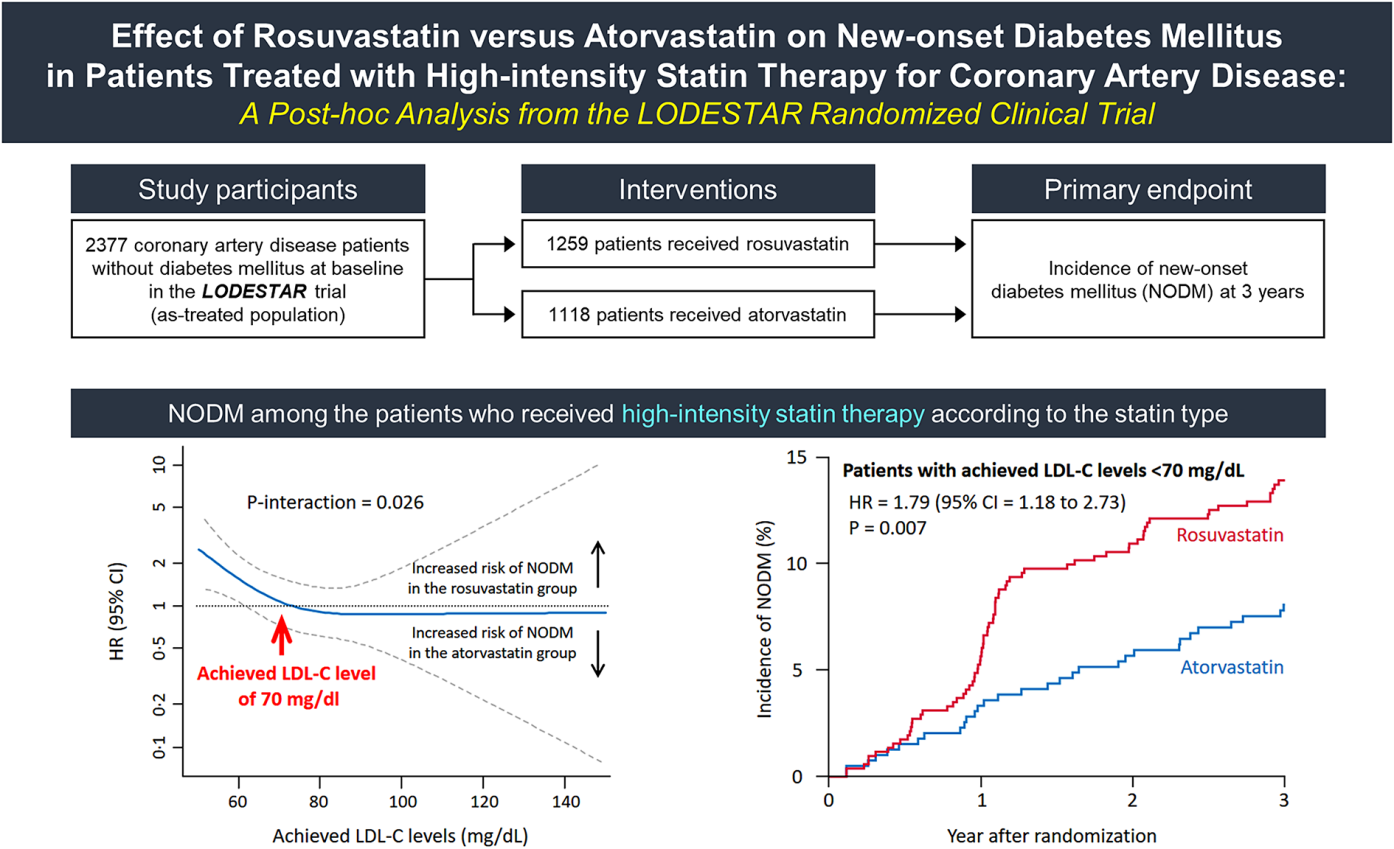

**Supplementary Information:**

The online version contains supplementary material available at 10.1186/s12933-024-02386-w.

## Introduction

For patients with coronary artery disease (CAD), intensive reduction of low-density lipoprotein cholesterol (LDL-C) levels via 3-hydroxy-3-methylglutarylcoenzyme A (HMG-CoA) reductase inhibitor (statin) therapy is recommended [1, 2]. However, statin use has been associated with increased risk for new-onset diabetes mellitus (NODM) [3–6]. An increased risk of NODM was more frequently observed in patients with higher-intensity statin therapy than in those with lower-intensity statin therapy [7]. While high-intensity statins are generally used as the initial choice for LDL-C lowering therapy in the secondary prevention of cardiovascular disease, only rosuvastatin and atorvastatin can provide high-intensity statin therapy [1, 2]. However, it remains uncertain whether the risk of NODM differs between rosuvastatin and atorvastatin. Recently, a safety endpoint in the LODESTAR (Low-density lipoprotein cholesterol-targeting statin therapy versus intensity-based statin therapy in patients with coronary artery disease) trial identified a higher incidence of NODM in patients receiving rosuvastatin than in those on atorvastatin [8, 9]. In the previous report, the NODM was only evaluated according to the population randomized (intention-to-treat population), rather than by what each patient actually received (as-treated population). In addition, questions may arise as to whether these findings are dependent on the lipid-lowering efficacy of the medication, as a significantly lower LDL-C level was observed in the rosuvastatin group than in the atorvastatin group.

Therefore, in this post-hoc analysis of the LODESTAR trial, we evaluated whether there is a difference in the incidence of NODM between rosuvastatin and atorvastatin in a head-to-head comparison with consideration of the type of statin that was actually given, particularly in patients treated with high-intensity statin therapy. We also assessed the comparative effect of rosuvastatin versus atorvastatin according to the achieved LDL-C levels.

## Methods

### Study design and participants

The LODESTAR trial was an investigator-initiated, multicenter, randomized trial conducted at 12 centers in South Korea. The protocol was approved by the institutional review board at each participating center. The study was performed according to the principles of the Declaration of Helsinki. The main outcomes of the LODESTAR trial were previously reported [8, 9]. Briefly, in the LODESTAR trial, patients with clinically diagnosed CAD underwent 2-by-2 factorial randomization according to: (1) the type of statin (rosuvastatin versus atorvastatin), and (2) the statin intensity maintenance strategy (treat-to-target strategy with target goal LDL-C levels versus high-intensity statin therapy without a target) [8, 9]. Details about the inclusion and exclusion criteria are provided in Additional file 1: Table S1. All participants provided written informed consent. In this post-hoc analysis evaluating the development of NODM during statin therapy, only participants without DM at baseline were included.

### Randomization and study procedures

Eligible patients were randomized in a 1:1 manner to receive either rosuvastatin or atorvastatin. In addition, as a factorial randomization, these participants were also randomized to receive a statin using either the targeted strategy of titrated-intensity statin therapy (treat-to-target strategy group) or the fixed strategy using high-intensity statin therapy (high-intensity statin strategy group). Web-response permuted-block randomization (mixed blocks of 4 or 6) was used at each participating site to allocate the patients. The patients were stratified by the presence of DM, baseline LDL-C levels ≥ 100 mg/dL, and acute coronary syndrome. The allocation sequence was computer-generated by an external programmer who was not involved in the trial. The physicians and research coordinators were able to access the web-response system.

The intensity of statin treatment was divided into three categories according to the 2018 American College of Cardiology/American Heart Association guidelines for the treatment of blood cholesterol [1]. In the treat-to-target strategy group, the target LDL-C level was below 70 mg/dL, and the statin intensity was titrated as follows. For statin-naïve patients, moderate-intensity statin therapy was initiated. For those who were already taking a statin, an equivalent intensity was maintained when LDL-C was below 70 mg/dL at randomization, and the intensity was up-titrated when LDL-C was ≥ 70 mg/dL. During follow-up, there was up-titration for those with LDL-C ≥ 70 mg/dL, maintenance of the same intensity for those with LDL-C ≥ 50 mg/dL to < 70 mg/dL, and down-titration for those with LDL-C < 50 mg/dL. In the high-intensity statin strategy group, high-intensity statin therapy was maintained without adjustment. In the LODESTAR trial, patients were treated with rosuvastatin 10 mg or atorvastatin 20 mg for moderate-intensity statin therapy, and rosuvastatin 20 mg or atorvastatin 40 mg for high-intensity statin therapy. For other medical treatments, guideline-directed medical therapy was strongly recommended.

Clinical and laboratory findings were assessed at baseline. All patients were scheduled for follow-up visits at 6 weeks and 3, 6, 12, 24, and 36 months. General health status, use of drugs, and the occurrence of clinical endpoints or adverse events were assessed at baseline and during each follow-up visit. The following results were followed serially at 6 weeks and 12, 24, and 36 months: lipid profiles, including total cholesterol, LDL-C, high-density lipoprotein cholesterol, and triglyceride levels. When the dose or type of study medication was changed during follow-up, patients were recommended to present for a laboratory test within 4 to 6 weeks. To monitor adverse effects related to the statin therapy, plasma glucose, hemoglobin A1c, aspartate aminotransferase, alanine aminotransferase, creatinine, and creatine kinase levels were assessed.

### Study endpoint

The primary endpoint of this study was the NODM, which was defined as a fasting plasma glucose level ≥ 126 mg/dL or new initiation of an antidiabetic drug according to the protocol [10, 11]. Firstly, the incidence of NODM was compared between rosuvastatin and atorvastatin in the intention-to-treat population. Secondly, the incidence of NODM was compared in the as-treated population, particularly with high-intensity statin therapy as the principal population of interest.

### Statistical analyses

Categorical data are presented as numbers (percentages). Continuous data are presented as mean ± standard deviation and median (interquartile range) for normal and skewed distribution, respectively. In the intention-to-treat population, all participants were included as randomly assigned to a treatment group. In the as-treated population, the participants who received ezetimibe in addition to statin therapy were excluded, as were those who received statins other than rosuvastatin or atorvastatin were excluded. Finally, the participants who actually received rosuvastatin monotherapy were termed the rosuvastatin group, and those who actually received atorvastatin monotherapy were termed the atorvastatin group. The intensities of the statin were also considered based on what the patients actually received.

The cumulative incidence of the primary endpoint at 3 years was estimated using Kaplan-Meier curves for a time-to-event analysis from the time of randomization to the occurrence of NODM development during follow-up. Hazard ratios (HRs) with 95% confidence intervals (CIs) were calculated using Cox regression analysis. Cox regression analyses with interaction tests were used to assess the differential therapy effects by the achieved LDL-C groups. A proportional hazard model, using restricted cubic splines with three knots, was developed to explore the association between NODM and achieved LDL-C levels as a continuous variable. The model was depicted graphically. Statistical analyses were conducted using R, version 4.3.1 (R Foundation). All tests were two-sided and statistical significance was set at *P* < 0.05.

## Results

### Participants

**Fig. 1:**
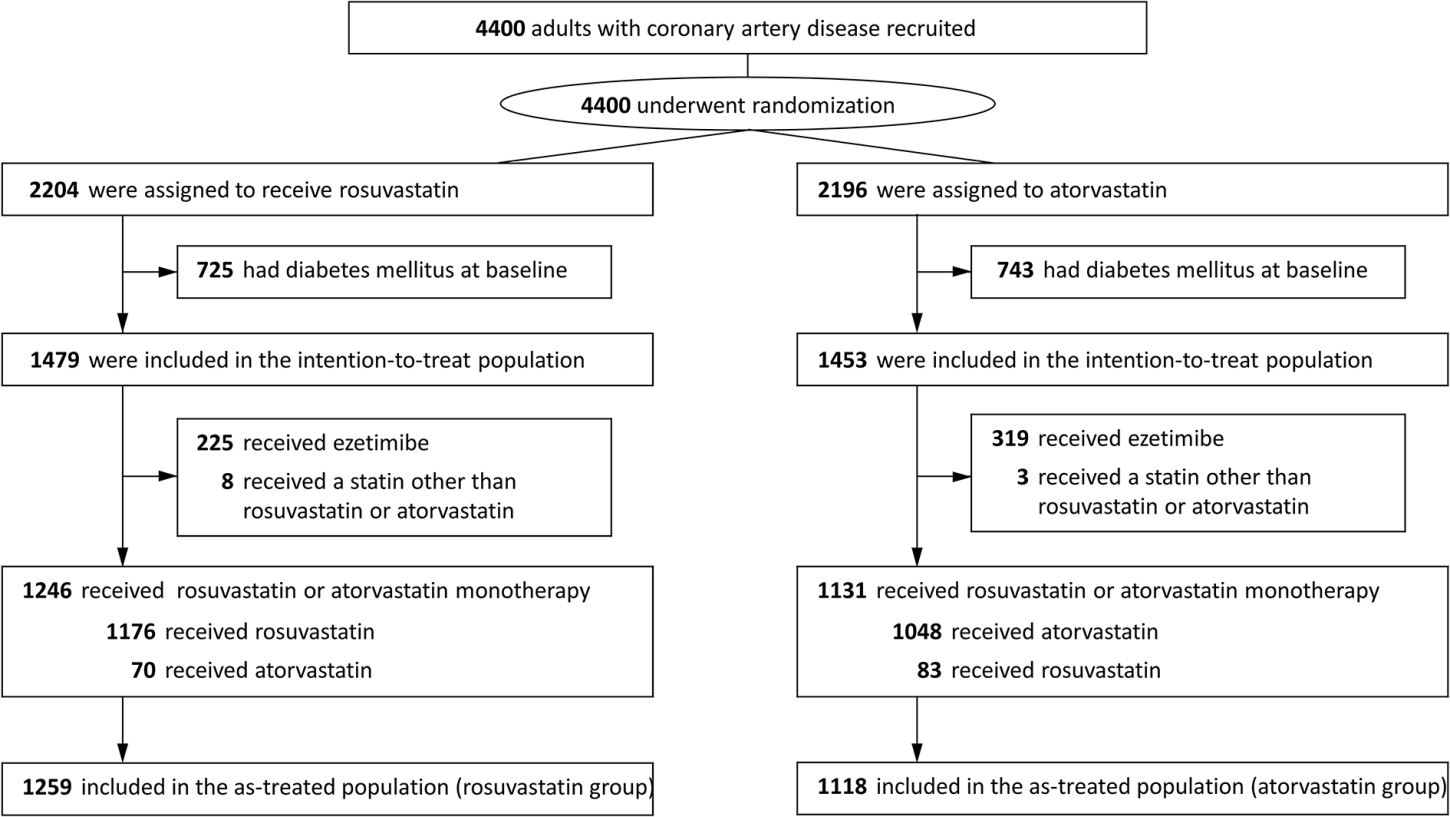
Study flow

Between September 2016 and November 2019, a total of 4400 patients were enrolled in the LODESTAR trial. Of these patients, 725 patients in the rosuvastatin group and 743 patients in the atorvastatin group were excluded because they had DM at baseline (Fig. 1). A total of 1479 patients in the rosuvastatin group and 1453 patients in the atorvastatin group were included in the intention-to-treat population. In the as-treated population, 544 patients who received ezetimibe in combination with statin therapy, and 11 patients who received other types of statins were excluded (Fig. 1). Finally, 2377 patients were analyzed in an as-treated population: 1259 (1176 plus 83) patients in the rosuvastatin group and 1118 (1048 plus 70) patients in the atorvastatin group. The baseline characteristics in the as-treated populations are shown in Additional file 1: Table S2. The baseline characteristics in the as-treated population with high-intensity statin therapy are presented in Table 1. The two groups were well balanced except the fasting glucose, and lipid lowering therapy before randomization.

**Table 1:** Baseline characteristics in the as-treated population with high-intensity statins

	Rosuvastatin *N* = 948	Atorvastatin *N* = 856	*P*-value
Age, mean (SD), years	65 ± 9	65 ± 10	0.623
Male	675 (71)	621 (73)	0.526
Female	273 (29)	235 (28)	0.526
Weight, mean (SD), kg	66 ± 10	67 ± 10	0.500
Height, mean (SD), cm	164 ± 8	165 ± 8	0.418
Body-mass index, mean (SD), kg/m^2^	24.6 (2.9)	24.6 (2.8)	0.953
Past medical history			
Hypertension	600 (63)	535 (63)	0.728
Chronic kidney disease	34 (4)	37 (4)	0.422
End-stage kidney disease on dialysis	1 (< 1)	1 (< 1)	0.942
Previous PCI	511 (54)	443 (52)	0.361
Previous CABG	55 (6)	46 (5)	0.693
Previous stroke	39 (4)	38 (4)	0.733
Current smoker	135 (14)	1119 (14)	0.836
Estimated GFR, mean (SD), ml/min/1.73 m^2^	90 ± 15	90 ± 15	0.989
Lipids, mean (SD), mg/dL			
Low-density lipoprotein cholesterol	91 ± 31	92 ± 31	0.582
High-density lipoprotein cholesterol	48 ± 12	48 ± 11	0.656
Total cholesterol	163 ± 38	162 ± 36	0.462
Triglycerides	132 ± 72	132 ± 79	0.787
Fasting glucose, mg/dL*			0.031
<99	381 (41)	306 (37)	
100–125	472 (51)	435 (52)	
≥126	75 (8)	94 (11)	
Clinical presentation at randomization			0.090
Acute myocardial infarction within 1 year	58 (6)	78 (9)	
> 1 year after myocardial infarction	121 (13)	118 (14)	
Unstable angina or revascularization within 1 year	182 (19)	148 (17)	
>1 year after unstable angina or revascularization	395 (42)	329 (38)	
Detection of CAD at screening without symptoms	192 (20)	183 (21)	
Lipid lowering therapy before randomization			
Statin^†^			0.004
High-intensity statin	206 (22)	245 (29)	
Moderate-intensity statin	549 (58)	465 (54)	
Low-intensity statin	16 (2)	8 (1)	
None	177 (19)	138 (16)	
Ezetimibe	79 (8)	58 (7)	0.212

CABG = coronary-artery bypass grafting; CAD = coronary artery disease; GFR = glomerular filtration rate; PCI = percutaneous coronary intervention

* 20 patients in the rosuvastatin group and 21 patients in the atorvastatin group did not measure fasting glucose at baseline

**†** The intensity of statin treatment was divided according to the 2018 American College of Cardiology/American Heart Association guideline for the treatment of blood cholesterol

### Achieved LDL-C levels

A mean achieved LDL-C level for 3 years was significantly lower in the rosuvastatin group than it was in the atorvastatin group in the intention-to-treat population (70.2 ± 20.8 versus 71.9 ± 18.7 mg/dL; *P* = 0.019) and in the as-treated population (68.2 ± 19.7 versus 71.6 ± 18.0 mg/dL; *P* < 0.001). The mean LDL-C levels and other lipid profiles during the follow-up in the as-treated population receiving high-intensity statin therapy are presented in Additional file 1: Table S3. In the as-treated population with high-intensity statin therapy, a mean achieved LDL-C level was also significantly lower in the rosuvastatin group than it was in the atorvastatin group (69.8 ± 19.6 versus 72.4 ± 18.0 mg/dL, *P* = 0.004).

### Development of NODM

In the intention-to-treat population, NODM developed in 152 patients among 1479 patients in the rosuvastatin group (10.4%) and in 119 patients among 1453 patients in the atorvastatin group (8.4%) (HR = 1.26, 95% CI = 0.99 to 1.60, *P* = 0.058) (Table 2). In the as-treated population, it was observed in 10.2% (127/1259) of the rosuvastatin group and 8.3% (91/1118) of the atorvastatin group (HR = 1.24, 95% CI = 0.95 to 1.63, *P* = 0.115) (Table 2). When the patients were classified according to statin intensity in the as-treated population, the incidence of NODM was not different between the two groups receiving low to moderate-intensity statins (6.9% versus 7.0%, HR = 0.98, 95% CI = 0.52 to 1.84, *P* = 0.948) (Table 2).

**Table 2:** New-onset diabetes mellitus (NODM) between rosuvastatin and atorvastatin treatment

	Rosuvastatin	Atorvastatin	HR (95% CI)	*P*-value
Intention-to-treat population	152/1479 (10.4)	119/1453 (8.4)	1.26 (0.99 to 1.60)	0.058
As-treated population*				
Overall patients	127/1259 (10.2)	91/1118 (8.3)	1.24 (0.95 to 1.63)	0.115
Low to moderate-intensity statin	21/290 (6.9)	18/244 (7.0)	0.98 (0.52 to 1.84)	0.948
High-intensity statin	106/948 (11.4)	73/856 (8.8)	1.32 (0.98 to 1.77)	0.071
Achieved LDL-C levels^†^				
< 70 mg/dL	71/517 (13.9)	31/398 (8.0)	1.79 (1.18 to 2.73)	0.007
≥ 70 mg/dL	35/431 (8.3)	42/458 (9.4)	0.87 (0.56 to 1.37)	0.549

Data are number of patients/total number of patients (%). CI = confidence interval; HR = hazard ratio; LDL-C = low-density lipoprotein cholesterol

* As-treated population was defined according to the actually received type of the statin after exclusion of those who received ezetimibe or a statin other than rosuvastatin or atorvastatin

**†** From the cubic spline analysis plotting, an increase of NODM in the rosuvastatin group versus the atorvastatin group began below an achieved LDL-C level of 70 mg/dL, which was determined as a cut-off value. An interaction test was performed between the type of statin (rosuvastatin versus atorvastatin) and the achieved LDL-C levels (< 70 versus ≥ 70 mg/dL). The P value for interaction was 0.022

**Fig. 2:**
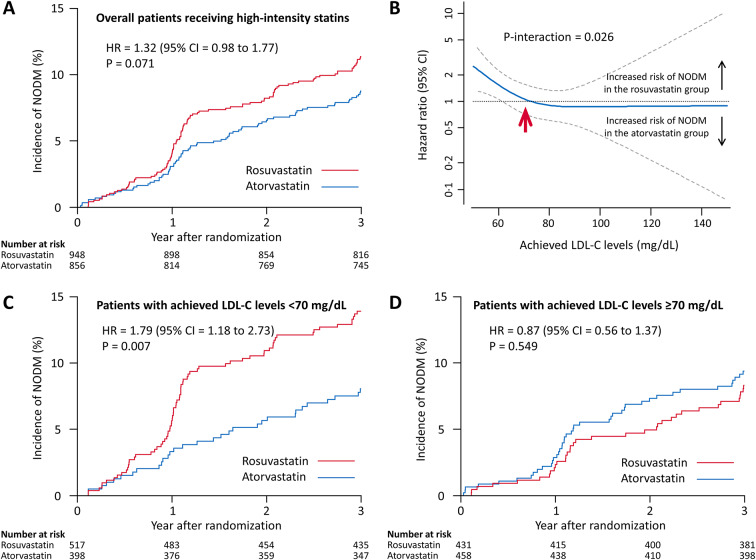
New-onset diabetes mellitus (NODM) among the patients who received a high-intensity statin according to the statin type.  **(A)** The incidence of NODM in overall patients receiving high-intensity statin therapy. **(B)** Cubic spline analysis of the risk of NODM in the rosuvastatin group versus atorvastatin group according to the achieved LDL-C levels. **(C)** The incidence of NODM in the patients with achieved LDL-C levels < 70 mg/dL. **(D)** The incidence of NODM in the patients with achieved LDL-C levels ≥ 70 mg/dL. From the cubic spline analysis plotting (**B**), an increase of NODM in the rosuvastatin group versus atorvastatin group began below an achieved LDL-C level of 70 mg/dL (red arrow), which was determined as a cut-off value. CI = confidence interval; HR = Hazard ratio; LDL-C = low-density lipoprotein cholesterol

In the subset of those who received high-intensity statin therapy, the incidence of NODM was not different between those who received rosuvastatin and those who received atorvastatin (11.4% versus 8.8%, HR 1.32, 95% CI = 0.98 to 1.77, *P* = 0.071) (Table 2 and Fig. 2A). Because the achieved mean LDL-C level was significantly lower in the rosuvastatin group than it was in the atorvastatin group, their effects on NODM were assessed according to the achieved LDL-C levels. Although the effect of rosuvastatin versus atorvastatin on NODM was consistent when the LDL-C was > 70 mg/dL, an increase of NODM in the rosuvastatin group versus the atorvastatin group began below an achieved LDL-C level of 70 mg/dL (P-interaction = 0.026) (Fig. 2B). The risk of NODM was significantly higher in patients on rosuvastatin than in those on atorvastatin among patients who achieved an LDL-C < 70 mg/dL (13.9% versus 8.0%, HR = 1.79, 95% CI = 1.18 to 2.73, *P* = 0.007). In contrast, the risk of NODM was not different between the two groups among patients who achieved LDL-C ≥ 70 mg/dL (8.3% versus 9.4%, HR = 0.87, 95% CI = 0.56 to 1.37, *P* = 0.549) (Fig. 2C and D, and Table 2). A significant interaction between the type of statin and the LDL-C level (< 70 versus ≥ 70 mg/dL) was also observed (P-interaction = 0.022).

## Discussion

In this post-hoc analysis from the LODESTAR trial, the incidence of NODM was not significantly different between rosuvastatin and atorvastatin when considering which high-intensity statin type was actually given (as-treated population). However, the risk of NODM according to the statin type appears to be dependent on the achieved LDL-C levelsWhen the achieved LDL-C level was < 70 mg/dL, the risk of NODM was higher in the rosuvastatin group than it was in the atorvastatin group, suggesting that there may be a drug effect related to statin type.

Although intensive reduction of LDL-C levels with statin therapy is recommended [1, 2], the increased risk of NODM with statin therapy has been a major concern for both physicians and patients. According to a meta-analyses of 13 statin trials, statin therapy was associated with a 9% increased risk for NODM [6]. In the LODESTAR trial, we previously reported a significantly higher incidence of NODM with rosuvastatin treatment compared to that with atorvastatin treatment as a safety endpoint [8]. However, this finding was observed in all patients without exclusion of those with DM at baseline. In addition, the incidence of NODM was evaluated according to the population as randomized. In this post-hoc analysis, NODM was assessed in the as-treated population, according to the type of statin that was actually given. The incidence of NODM was numerically higher in the rosuvastatin group than it was in the atorvastatin group, but it did not achieve statistical significance. Because the achieved LDL-C level was significantly lower in the rosuvastatin group than it was in the atorvastatin group, we also assessed the risk of NODM by the statin type according to the achieved LDL-C levels. We found that there is a significant interaction between the statin type and the achieved LDL-C levels for NODM. This result suggests that the risk of NODM by statin type may be partly attributed to the LDL-C lowering efficacy of the statin therapy. Although the mechanisms of statin therapy and NODM are not yet fully understood, a meta-analysis of genetic data from 43 studies revealed that the association could be related to the reduced activity of HMG-CoA reductase, which is the target of statin therapy [12]. Two single-nucleotide polymorphisms, rs17238484-G and rs12916-T, in the HMG-CoA reductase gene were found to lower LDL-C levels by 2.3 mg/dL and increase the risk of NODM by 2% and 6%, respectively [12]. To the extent that the risk of NODM is associated with the level of inhibition of HMG-CoA reductase activity, lower LDL-C levels—indicating stronger inhibition of HMG-CoA reductase—may also contribute to the higher incidence of NODM with rosuvastatin, which has a greater binding affinity for HMG-CoA reductase than atorvastatin. [3, 13]. However, it is unclear whether NODM is purely a statin-associated side effect or is simply associated with lowering LDL-C and would be present with the use of other lipid-lowering agents [14]. A meta-analysis of randomized clinical trials with statins and statin/proprotein convertase subtilisin-kexin type 9 (PCSK9) inhibitors use in 163,688 nondiabetic patients showed no significant association between LDL-C reduction and NODM incidence [15] However, a sub-study of JUPITER (Justification for the Use of Statins in Prevention: an Intervention Trial Evaluating Rosuvastatin) trial demonstrated that rosuvastatin-treated patients attaining LDL-C < 30 mg/dL were at increased risk for developing NODM than did those with LDL-C ≥ 30 mg/dL [16]. A Mendelian randomization study also demonstrated that variants in PCSK9 and HMG-CoA reductase genes were correlated with higher diabetes risk per unit decrease in LDL-C [17].

In this study, when the LDL-C was lowered to < 70 mg/dL with rosuvastatin, the risk of NODM increased more than when the same was achieved with atorvastatin. Recent pairwise, network, and dose-response meta-analyses aimed to evaluate how the associations vary by statin type and adverse events; however, these analyses only included patients being treated for primary prevention of cardiovascular disease, and also only indirect comparisons were possible [18]. For comparisons between the different statin type, atorvastatin (HR = 1.49, 95% CI = 1.08 to 2.05) and rosuvastatin (HR = 1.50, 95% CI = 1.16 to 1.94) had a higher risk of NODM than did pitavastatin, although there were no other significant differences between the types of statins, including in the comparison of rosuvastatin and atorvastatin [18]. In both primary and secondary prevention, it is important to understand the adverse effects of statin therapy. This is particularly true regarding NODM, as it is dependent on the dosage or intensity of the statin therapy. In a meta-analysis of 5 trials, NODM more frequently developed in patients receiving higher-intensity statin therapy than it did in those on lower-intensity statin therapy [7]. Another meta-analyses also assessed NODM development according to different types and doses of statins [19]. There was a gradient for NODM risk across different statin types and doses. Pravastatin 40 mg was associated with the lowest rate of NODM (odds ratio [OR] = 1.07; 95% CI = 0.86 to 1.30), whereas rosuvastatin 20 mg was associated with the highest numeric incidence of NODM (OR = 1.25; 95% CI = 0.82 to 1.90), and atorvastatin 80 mg was intermediate (OR = 1.15; 95% CI = 0.90 to 1.50) [19]. However, in that analysis, there was no direct comparison between rosuvastatin and atorvastatin. On the other hand, this post-hoc analysis of the LODESTAR trial directly compared the incidence of NODM between rosuvastatin and atorvastatin in patients requiring high-intensity statin therapy for secondary prevention. We suggest that the choice of the statin type should be determined considering the achieved LDL-C levels, especially when individuals are at increased risk of NODM, such as prediabetes. However, the exact mechanism by which NODM varies by statin type remains unclear. Therefore, our results should be interpreted cautiously.

This study has several limitations. First, this was a post-hoc analysis, although NODM was the main secondary safety endpoint in the LODESTAR trial. Second, the definition of NODM did not include oral glucose tolerance tests, random plasma glucose measurements, or hemoglobin A1c levels. However, the definition was pre-specified in the protocol. Third, the follow-up duration may have been too short to reflect the long-term effects of the two statin types, particularly regarding NODM development. Fourth, the total duration of statin treatment before randomization was not considered. Therefore, our findings need to be considered only as hypothesis-generating, and further dedicated investigation with longer follow-up is warranted.

## Conclusions

In this post-hoc analysis of the LODESTAR trial, the incidence of NODM was not significantly different between rosuvastatin and atorvastatin among CAD patients on high-intensity statin therapy. However, it appears that the risk of NODM according to the statin types may be affected by the efficacy of LDL-C lowering. The risk of NODM was significantly higher in the rosuvastatin group than in the atorvastatin group when the achieved LDL-C level was < 70 mg/dL. However, the risk of NODM did not differ between the two groups when the achieved LDL-C level was LDL-C ≥ 70 mg/dL.

### Electronic supplementary material

Supplementary Material 1

## Data Availability

The data regarding this article will be shared by the corresponding author upon reasonable request.

## Abbreviations

CAD: Coronary artery disease
CI: Confidence interval
HMG-CoA: 3-hydroxy-3-methylglutarylcoenzyme A
HR: Hazard ratio
LDL-C: Low-density lipoprotein cholesterol
NODM: New-onset diabetes mellitus
OR: Odds ratio
