# Supplementary material for: Effect of rosuvastatin versus atorvastatin on new-onset diabetes mellitus in patients treated with high-intensity statin therapy for coronary artery disease: a post-hoc analysis from the LODESTAR randomized clinical trial
Source: Cardiovasc Diabetol. 2024 Aug 7;23:287. doi: 10.1186/s12933-024-02386-w (PMC11304915; doi:10.1186/s12933-024-02386-w)
Supplement: Supplementary file 1 — Supplementary Material 1 [file 12933_2024_2386_MOESM1_ESM.docx]

**Additional file 1**

**Effect of Rosuvastatin versus Atorvastatin on New-onset Diabetes Mellitus in Patients Treated with High-intensity Statin Therapy for Coronary Artery Disease: A Post-hoc Analysis from the LODESTAR Randomized Clinical Trial**

**Contents**

**I. The LODESTAR Investigators** ∙∙∙∙∙∙∙∙∙∙∙∙∙∙∙∙∙∙∙∙∙∙∙∙∙∙∙∙∙∙∙∙∙∙∙∙∙∙∙∙∙∙∙∙∙∙∙∙∙∙∙∙∙∙∙∙∙∙∙∙∙∙∙∙∙∙∙∙∙∙∙∙∙∙∙∙∙∙∙∙ 2

**II. Supplementary Tables**

**Table S1**. Inclusion and exclusion criteria ∙∙∙∙∙∙∙∙∙∙∙∙∙∙∙∙∙∙∙∙∙∙∙∙∙∙∙∙∙∙∙∙∙∙∙∙∙∙∙∙∙∙∙∙∙∙∙∙∙∙∙∙∙∙∙∙∙∙∙∙∙∙∙∙∙∙∙∙∙∙∙∙∙∙∙∙∙∙ 3

**Table S2**. Baseline characteristics in the as-treated population ∙∙∙∙∙∙∙∙∙∙∙∙∙∙∙∙∙∙∙∙∙∙∙∙∙∙∙∙∙∙∙∙∙∙∙∙∙∙∙∙∙∙∙∙∙∙∙ 4

**Table S3**. Low-density lipoprotein cholesterol levels and other lipid profile in the as-treated population receiving high-intensity statin therapy ∙∙∙∙∙∙∙∙∙∙∙∙∙∙∙∙∙∙∙∙∙∙∙∙∙∙∙∙∙∙∙∙∙∙∙∙∙∙∙∙∙∙∙∙∙∙∙∙∙∙∙∙∙∙∙∙∙∙∙∙∙∙∙∙∙∙ 5

**The LODESTAR Investigators**

Myeong-Ki Hong, Donghoon Choi, Young-Guk Ko, Byeong-Keuk Kim, Jung-Sun Kim, Chul-Min Ahn, Sung-Jin Hong, Seung-Jun Lee, Yong-Joon Lee (Severance Hospital, Yonsei University College of Medicine); Bum-Kee Hong, Hyuck Moon Kwon, Jong-Youn Kim, Pil Ki Min, Young Won Yoon, Byoung Kwon Lee, Se-Joong Rim, Eui-Young Choi (Gangnam Severance Hospital); Woong Chol Kang, Pyung Chun Oh (Gachon University College of Medicine); Jong-Young Lee (Kangbuk Samsung Hospital, Sungkyunkwan University School of Medicine); Jin-Bae Lee, Kee Sik Kim, Ji Yong Choi, Jae Kean Ryu, Seung Pyo Hong, Chang Yeon Kim (Daegu Catholic University Medical Center); Tae-Hyun Yang, Hyung-Jin Cho (Inje University Busan Paik Hospital); Junghan Yoon, Min-Soo Ahn, Sung Gyun Ahn, Jun-Won Lee, Jung-Woo Son (Wonju Severance Christian Hospital); Yangsoo Jang (CHA University College of Medicine); Hyuck-Jun Yoon, Cheol Hyun Lee, Jongmin Hwang, Yun-Kyeong Cho, Seung-Ho Hur, Seongwook Han, Chang-Wook Nam, Hyoungseop Kim, Hyoung-Seob Park, In-Cheol Kim (Keimyung University Dongsan Medical Center); Yun-Hyeong Cho, Hyeon-Ju Jeong, Jin-Ho Kim, Lim Chewan, Yongsung Suh, Eui Seok Hwang, Ji Hyun Lee (Myongji Hospital, Hanyang University College of Medicine); Sung Yun Lee, Sung Uk Kwon (Inje University Ilsan Paik Hospital); Song-Yi Kim (Jeju National University Hospital); Keun-Ho Park, Hyun Kuk Kim (Chosun University Hospital)

**Table S1. Inclusion and exclusion criteria**

| **Inclusion Criteria**   - Patients ≥19 years old - Patients clinically diagnosed with coronary artery disease: stable angina, unstable angina, acute non-ST elevation myocardial infarction, and acute ST elevation myocardial infarction - Patients with signed informed consent   **Exclusion Criteria**   - Pregnant women or women with potential childbearing during the study period - Patients with severe adverse events or hypersensitive to statin - Patients receiving drug that interacts with statin (strong inhibitor of cytochrome p-450 3A4 or 2C9) - Patients with risk factors for myopathy, hereditary muscle disorder, hypothyroidism, alcohol use disorder, severe hepatic dysfunction (3 times the normal reference values), or rhabdomyolysis - Life expectancy <3 years - Patients who could not be followed for more than 1 year - Patients who could not understand the consent form |
| --- |

**Table S2. Baseline characteristics in the as-treated population**

|  | **Rosuvastatin**  **N=1259** | **Atorvastatin**  **N=1118** | **P-value** |
| --- | --- | --- | --- |
| Age, mean (SD), years | 65±10 | 65±10 | 0.969 |
| Male | 894 (71) | 800 (72) | 0.768 |
| Female | 365 (29) | 318 (28) | 0.768 |
| Weight, mean (SD), kg | 66±10 | 67±10 | 0.554 |
| Height, mean (SD), cm | 164±8 | 165±8 | 0.207 |
| Body-mass index, mean (SD), kg/m^2^ | 24.5 (2.9) | 24.5 (2.7) | 0.696 |
| Past medical history |  |  |  |
| Hypertension | 799 (64) | 697 (62) | 0.573 |
| Chronic kidney disease | 44 (4) | 55 (5) | 0.083 |
| End-stage kidney disease on dialysis | 2 (<1) | 2 (<1) | 0.905 |
| Previous PCI | 674 (54) | 570 (51) | 0.214 |
| Previous CABG | 64 (5) | 54 (5) | 0.777 |
| Previous stroke | 61 (5) | 48 (4) | 0.521 |
| Current smoker | 177 (14) | 156 (14) | 0.941 |
| Estimated GFR, mean (SD), ml/min/1.73 m^2^ | 90±15 | 90±15 | 0.652 |
| Lipids, mean (SD), mg/dL |  |  |  |
| Low-density lipoprotein cholesterol | 88±32 | 90±32 | 0.387 |
| High-density lipoprotein cholesterol | 48±12 | 48±11 | 0.737 |
| Total cholesterol | 160±38 | 160±37 | 0.916 |
| Triglycerides | 131±73 | 134±89 | 0.451 |
| Fasting glucose, mean (SD), mg/dL* |  |  | 0.054 |
| <99 | 502 (41) | 400 (37) |  |
| 100 - 125 | 623 (51) | 573 (53) |  |
| ≥126 | 107 (9) | 119 (11) |  |
| Clinical presentation at randomization |  |  | 0.038 |
| Acute myocardial infarction within 1 year | 80 (6) | 95 (9) |  |
| >1 year after myocardial infarction | 155 (12) | 145 (13) |  |
| Unstable angina or revascularization within 1 year | 253 (20) | 191 (17) |  |
| >1 year after unstable angina or revascularization | 506 (40) | 419 (38) |  |
| Detection of CAD at screening without symptoms | 265 (21) | 268 (24) |  |
| Lipid lowering therapy before randomization |  |  |  |
| Statin**†** |  |  | 0.004 |
| High-intensity statin | 241 (19) | 270 (24) |  |
| Moderate-intensity statin | 725 (58) | 619 (55) |  |
| Low-intensity statin | 29 (2) | 12 (1) |  |
| None | 264 (21) | 217 (19) |  |
| Ezetimibe | 108 (9) | 79 (7) | 0.172 |

CABG=coronary-artery bypass grafting; CAD=coronary artery disease; GFR=glomerular filtration rate; PCI=percutaneous coronary intervention.

* 27 patients in the rosuvastatin group and 26 patients in the atorvastatin group did not measure fasting glucose at baseline.

**†** The intensity of statin treatment was divided according to the 2018 American College of Cardiology/American Heart Association guideline for the treatment of blood cholesterol.

**Table S3. Low-density lipoprotein cholesterol levels and other lipid profile in the as-treated population receiving high-intensity statin therapy**

|  | **Rosuvastatin**  **N=948** | **Atorvastatin**  **N=856** | **P-value** |
| --- | --- | --- | --- |
| **At 6 weeks** |  |  |  |
| Low-density lipoprotein cholesterol, mg/dL | 66.3±19.7 | 69.5±18.2 | 0.002 |
| High-density lipoprotein cholesterol, mg/dL | 46.9±11.6 | 47.3±11.3 | 0.231 |
| Total cholesterol, mg/dL | 135.4±26.9 | 136.4±28.0 | 0.724 |
| Triglycerides, mg/dL | 128.1±60.7 | 128.7±74.6 | 0.882 |
| **At 3 months** |  |  |  |
| Low-density lipoprotein cholesterol, mg/dL | 69.5±19.6 | 70.5±21.5 | 0.714 |
| High-density lipoprotein cholesterol, mg/dL | 46.9±11.6 | 47.3±11.3 | 0.320 |
| Total cholesterol, mg/dL | 134.9±26.4 | 137.7±27.8 | 0.307 |
| Triglycerides, mg/dL | 130.6±73.8 | 145.9±92.6 | 0.082 |
| **At 6 months** |  |  |  |
| Low-density lipoprotein cholesterol, mg/dL | 67.2±19.6 | 72.0±20.3 | 0.001 |
| High-density lipoprotein cholesterol, mg/dL | 47.3±11.5 | 47.6±11.5 | 0.700 |
| Total cholesterol, mg/dL | 135.3±25.4 | 140.5±30.4 | 0.008 |
| Triglycerides, mg/dL | 126.4±67.8 | 134.2±71.7 | 0.118 |
| **At 1 year** |  |  |  |
| Low-density lipoprotein cholesterol, mg/dL | 68.9±20.6 | 72.4±20.3 | 0.001 |
| High-density lipoprotein cholesterol, mg/dL | 47.5±11.5 | 47.8±11.7 | 0.571 |
| Total cholesterol, mg/dL | 136.5±26.6 | 137.3±28.0 | 0.589 |
| Triglycerides, mg/dL | 126.0±67.6 | 132.7±75.2 | 0.070 |
| **At 2 years** |  |  |  |
| Low-density lipoprotein cholesterol, mg/dL | 67.5±19.8 | 71.2±18.3 | <0.001 |
| High-density lipoprotein cholesterol, mg/dL | 47.4±11.6 | 47.3±11.4 | 0.870 |
| Total cholesterol, mg/dL | 135.7±26.6 | 137.4±24.9 | 0.233 |
| Triglycerides, mg/dL | 123.3±61.0 | 124.3±65.7 | 0.757 |
| **At 3 years** |  |  |  |
| Low-density lipoprotein cholesterol, mg/dL | 68.5±21.8 | 72.1±19.9 | 0.003 |
| High-density lipoprotein cholesterol, mg/dL | 46.8±11.3 | 47.1±11.8 | 0.708 |
| Total cholesterol, mg/dL | 135.8±25.6 | 136.7±26.2 | 0.530 |
| Triglycerides, mg/dL | 128.4±77.2 | 127.8±78.0 | 0.885 |
